# HER-2, p53, p21 and hormonal receptors proteins expression as predictive factors of response and prognosis in locally advanced breast cancer treated with neoadjuvant docetaxel plus epirubicin combination

**DOI:** 10.1186/1471-2407-7-36

**Published:** 2007-02-26

**Authors:** Daniel G Tiezzi, Jurandyr M Andrade, Alfredo Ribeiro-Silva, Fábio E Zola, Heitor RC Marana, Marcelo G Tiezzi

**Affiliations:** 1 Department of Gynecology and Obstetrics – Breast Disease Division – Ribeirao Preto School of Medicine – HCFMRP- USP, Av Bandeirantes, 3900, 14048-900 Ribeirão Preto, São Paulo, Brazil; 2 Department of Pathology – Ribeirao Preto School of Medicine – HCFMRP- USP, Ribeirao Preto, Brazil; 3 Colaborating Pathologist from Presidente Prudente Laboratory of Pathology and Cytopathology, S/A, Presidente Prudente, Brazil

## Abstract

**Background:**

Neoadjuvant chemotherapy has been considered the standard care in locally advanced breast cancer. However, about 20% of the patients do not benefit from this clinical treatment and, predictive factors of response were not defined yet. This study was designed to evaluate the importance of biological markers to predict response and prognosis in stage II and III breast cancer patients treated with taxane and anthracycline combination as neoadjuvant setting.

**Methods:**

Sixty patients received preoperative docetaxel (75 mg/m^2^) in combination with epirubicin (50 mg/m^2^) in *i.v*. infusion in D1 every 3 weeks after incisional biopsy. They received adjuvant chemotherapy with CMF or FEC, attaining axillary status following definitive breast surgery. Clinical and pathologic response rates were measured after preoperative therapy. We evaluated the response rate to neoadjuvant chemotherapy and the prognostic significance of clinicopathological and immunohistochemical parameters (ER, PR, p51, p21 and HER-2 protein expression). The median patient age was 50.5 years with a median follow up time 48 months after the time of diagnosis.

**Results:**

Preoperative treatment achieved clinical response in 76.6% of patients and complete pathologic response in 5%. The clinical, pathological and immunohistochemical parameters were not able to predict response to therapy and, only HER2 protein overexpression was associated with a decrease in disease free and overall survival (*P *= 0.0007 and *P *= 0.003) as shown by multivariate analysis.

**Conclusion:**

Immunohistochemical phenotypes were not able to predict response to neoadjuvant chemotherapy. Clinical response is inversely correlated with a risk of death in patients submitted to neoadjuvant chemotherapy and HER2 overexpression is the major prognostic factor in stage II and III breast cancer patients treated with a neoadjuvant docetaxel and epirubicin combination.

## Background

Neoadjuvant or primary chemotherapy in large primary breast cancer has been used with the purpose of reducing tumor volume and promoting less aggressive surgery [1]. The disease's downstaging promotes higher conservative breast surgery rates and [2], in patients with objective clinical and pathological response, an increase in disease free and overall survival rates has been observed [3,4]. Otherwise, about 10% – 35% of the patients do not benefit from this clinical approach [5,6]. The identification of this group of patients could avoid the chemotherapy side effects and point them toward other therapeutic alternatives.

Clinical and pathological criteria neither predict response to neoadjuvant chemotherapy nor accurately define tumor biology. Tumors with the same histological type, grade and clinical staging behave differently [7]. The current hypothesis is that morphologically similar tumors have distinct gene expression with distinct phenotypic profiles [8]. The development of new therapies directed at specific molecular expression has lead researchers to evaluate the usefulness of tumor protein expression as predictive factors of response.

Docetaxel, an agent that promotes microtubule stabilization and prevents depolymerization, can cause cell cycle arrest in the mitotic phase [9], and epirubicin, an anthracycline that interacts with topoisomerase II and blocks DNA transcription [10], have both currently been used in adjuvant and neoadjuvant breast cancer treatment. In a neoadjuvant setting, the combination of these two agents is among the most actively described chemotherapy regimens [11]. The study of the molecular tumor characteristics and effects of the taxane and anthracycline combination on protein modulation can provide information about the mechanism of action and play an important role in the individualization of neoadjuvant therapy.

We evaluated the expression of p53, p21 and HER-2 protein expression by immunohistochemistry (IHC) before and after neoadjuvant docetaxel plus epirubicin in locally advanced breast cancer patients and their relationship with clinical response and outcome.

## Methods

### Characteristics of patients and description of neoadjuvant therapy

Sixty women with a histological diagnosis of invasive breast cancer in incisional biopsy specimens were enrolled consecutively in the study. Inclusion criteria included patients with locally advanced epithelial breast cancer (AJCC clinical stage IIa, if T> 3 cm; IIb and III), Karnofski performance status greater than 70% and age under 75 years. From February 2000 to December 2002, patients received treatment with taxane and anthracycline (docetaxel 75 mg/m^2 ^and epirubicin 50 mg/m^2^, *i.v*. infusion, for one day every three weeks). No dose modification was allowed. The median cycle of chemotherapy was 3 cycles (2 – 5) and the patients' median age was 50.5 years (29 – 68). Thirty-one patients were at stage II and twenty-nine were at stage III. Presence of primary inflammatory breast carcinoma was an exclusion factor and invasive ductal carcinoma was the most common histological type (82.7%). Sixteen tumors were grade I (23%) and grade II and III was observed in 55.7% and 21.3% of the tumors, respectively. The median follow up time was 48 months (24 – 69). Table 1 summarizes the patients' characteristics.

None of these patients had distant metastasis nor had they received prior therapy for cancer. Hepatic and renal function tests and blood cell count were carried out at each chemotherapy cycle, and no toxicity greater than 1 or 2 was observed. Table 2 describes the toxicity effects due to neoadjuvant chemotherapy. Chemotherapy was continued until tumors became operable in primary inoperable lesions or until breast conserving surgery could be performed in primary operable tumors. The patients with primary inoperable lesions who had no clinical response, defined after at least two cycles of chemotherapy, were subjected to a new incisional biopsy and given a second line of treatment consisting of breast radiation therapy (total dose of 50 Gy) combined with a CMF chemotherapy regimen (cyclophosphamide 500 mg/m^2^, methotrexate 50 mg/m^2 ^and 5-fluoruracil 500 mg/m^2 ^*i.v*. infusion, for 1 day, each 21/21 days) before definitive breast surgery. All procedures were carried out with the prior informed consent of the patient.

### Adjuvant treatment

Adjuvant chemotherapy was offered to all patients. Regarding axillary lymph node status. The FEC regimen (5-fluoruracil 500 mg/m^2^, epirubicin 50 mg/m^2 ^and cyclophosphamide 500 mg/m^2 ^*i.v*. infusion, for 1 day, each 21/21 days) was administered when more than three positive axillary lymph nodes (ALN+) were present after neoadjuvant treatment. The CMF regimen (cyclophosphamide 500 mg/m^2^, methotrexate 50 mg/m^2 ^and 5-fluoruracil 500 mg/m^2 ^*i.v*. infusion, for 1 day, each 21/21 days) was used if there were three or less ALN+. All patients received a total of nine chemotherapy cycles. Hormonal adjuvant therapy was offered to all patients with estrogen or progesterone receptor positive tumors. Patients received tamoxifen at 20 mg PO for 60 months or until disease recurrence.

Adjuvant radiotherapy was performed in the residual breast parenchyma of all breast conserving surgery patients (50 Gy). Patients who underwent mastectomy received chest wall adjuvant radiotherapy if residual tumors were five centimeters or greater (at the highest pathological diameter) or if there was skin or muscle and chest wall invasion (50 Gy). The supraclavicular fossa was irradiated if there were four or more ALN+.

This study was approved by the local Ethics Committee in accordance with the ethical guidelines of the 1975 Declaration of Helsinki, revised 1983.

### Assessment of response to neoadjuvant chemotherapy

The pathological and clinical assessment of response to neoadjuvant chemotherapy was taken. All surgical specimens from the definitive breast procedure were submitted for pathological evaluation and were classified as having a complete response (pCR) when no residual invasive carcinoma was seen.

The clinical stage and size of the primary tumor was recorded before treatment. The measurement of the primary tumor consisted of the product of its greatest diameter and its perpendicular diameter. The clinical response was evaluated at each cycle of chemotherapy and prior to definitive surgery on day 21 of the last cycle of chemotherapy according to the product of primary tumor diameters and the axillary clinical status, and was classified as a complete response (CR), partial response (PR), stable disease (SD), or progressive disease (PD) according to standard UICC (International Union Against Cancer) criteria [12]. CR was defined as the disappearance of all clinical evidence of the tumor including the axillary site; PR was defined as a reduction of 50% or more in the sum of the products of measured lesions or an estimated decrease in tumor size of at least 50%, without the appearance of new lesions. Clinical down staging in axillary status was necessary; SD was defined as a decrease of less than 50% in the sum of the products of measured lesions, or an estimated decrease of less than 50% in lesion size or an increase of less than 25%, without the appearance of new lesions. Any measured or estimated increase greater than 25% or the appearance of new lesions was defined as PD. No change in clinical axillary status was classified as SD regardless the change in primary tumor diameter. The patients were classified into two groups: the objective response group (OR), into which all patients classified as CR or PR were placed, and the no response group (NR), containing all patients classified as SD or PD.

### Immunohistochemistry

All tissue samples had been routinely fixed in 4% neutral formalin and embedded in paraffin. Briefly, 3-μm-thick sections were cut from paraffin blocks containing representative tumor samples. Paraffin sections were de-waxed in xylene, rehydrated through a series of graded alcohols, placed in 10 mM citrate buffer and submitted to heat retrieval using a vapor lock for 40 minutes. After heating, the slides were allowed to cool to room temperature and briefly washed with Tris-buffered saline. Endogenous peroxidase activity was blocked with 3% hydrogen peroxide in methanol for 5 minutes. Normal serum (Novostain Super ABC kit, Novocastra, Newcastle upon Tyne, UK) was used for 30 minutes in order to block non-specific immunoassaying. Immunohistochemical staining was performed using an avidin-biotin peroxidase system (Novostain Super ABC kit, Novocastra, UK). The following primary antibodies were incubated overnight at room temperature: ER (1:100, clone 6F11, Novocasta, UK), PR (1:100, clone 1A6, Novocasta, UK), p21 (1:50, clone 4D10, Novocastra, UK), HER-2 oncoprotein (1:300, clone CB11, Novocastra, UK), and p53 (1:100, clone DO-7, Novocastra, UK). Following washes in PBS, biotinylated universal secondary antibody (Novostain Super ABC kit, Novocastra, UK) was applied for 30 minutes. The sections were incubated with the avidin-biotin complex reagent (Novostain Super ABC kit, Novocastra, UK) for 30 minutes and developed with 3.3-diaminobenzidine tetrahydrochloride (DAB) in phosphate-buffered saline, pH 7.5, containing 0.036% hydrogen peroxide for 5 minutes. Light Mayer's hematoxylin was applied as a counterstain. The slides were then dehydrated in a series of ethanols and mounted with Permount (Fischer, Fairlawn, NJ).

Cases of invasive ductal carcinoma, previously known to be positive for ER, PR, p21, p53 or HER-2, were used as positive controls. Negative controls for immunostaining were prepared by omission of the primary antibody.

### Scoring methods

The cases were interpreted as positive for p21 if more than 5% of the tumors cells showed nuclear staining [13] and were considered positive to ER, PR and p53 protein expression if more than 10% of the tumors cells showed nuclear staining. HER-2 expression was scored according to the degree and the proportion of membrane staining according to HercepTest protocol [14,15]. HER-2 expression was negative with a score of 0 or 1+. A score of 0 was defined as no staining or membrane staining in less than 10% of tumor cells. A score 1+ comprised faint or partially-stained membrane in more of 10% of tumor tissue. Overexpression of HER-2 was scored as 2+ when weak to moderate complete membrane staining was present in more than 10% of tumor cells. A score of 3+ was interpreted as strong, complete membrane staining in more than 10% of the tumor.

### Study endpoints

The primary goal was to test the correlation among p53, p21 and HER-2 protein expression and clinical response to neoadjuvant chemotherapy with docetaxel and epirubicin combination and to evaluate the impact of immunohistochemical and clinic-pathological parameters in disease free and overall survivals. A secondary goal was to study the ability of neoadjuvant taxane and anthracycline based chemotherapy to change tumor protein expression. We also studied the association between protein changes and the impact in disease free and overall survivals. The third objective was to evaluate the efficacy and toxicity of a neoadjuvant docetaxel and epirubicin combination. To test the treatment efficacy, we examined a clinical parameter of response and the type of surgery performed.

### Statistical analysis

The McNemar test was used to evaluate the paired correlation between p53, p21 and HER-2 protein expression before and after neoadjuvant chemotherapy. Spearman's coefficient of rank correlation [16] was used to determine the correlation between axillary lymph node status and clinical response and to evaluate the correlation between clinical response and the risk of death in disease progression. The relationship between the clinical response with the patients' characteristics and histopathological patterns was evaluated with Fisher exact test and chi-squared test. The relationship between the number of chemotherapy cycles was analyzed with the Mann-Whitney test. The disease free survival (DFS) and overall survival (OS) were analyzed by the Kaplan-Meyer curve. MedCalc version 6.16.000 and Graphpad Prism version 4 software were used for the above statistical analyses. Multivariate analysis, to test the correlation between HER-2 protein expression, clinical response, axillary lymph node status and type of surgery performed with the risk of recurrence and death to disease progression, was performed using a nominal logistic fit with JMP 6.0 SAS software. The statistical significance was established as *p *< 0.05.

## Results

### Neoadjuvant treatment, clinical response rate and surgery

The objective clinical response (OR) was observed in 46 patients (76.6%) treated with neoadjuvant chemotherapy. The clinical complete response rate (cCR) was 15% (nine patients) and the pathological complete response rate (pCR), defined as the absence of residual invasive carcinoma, was 5% (three patients). Patients with stable and progressive disease were defined as no response patients (NR). The NR rate was 23.3% (14 patients) and progressive disease was observed in only two patients (3.3%).

Surgery could be performed in fifty-nine patients (98.3%). One patient with no response to the primary docetaxel plus epirubicin combination was submitted to second line chemo-radiation therapy. The tumor was resistant to second line treatment and she died due to metastatic disease progression before surgery could be performed. Breast conserving surgery could be performed in forty patients (66.7%). Modified Radical Mastectomy was performed in patients for whom breast-conserving therapy was not indicated. Patients with OR had a higher breast conserving surgery rate than patients with NR (73.9% *vs*. 35.7%; *p *= 0.03, chi-square test).

Axillary lymph nodes were assessed histologicaly in 59 patients undergoing axillary node dissection. The median number of positive lymph nodes in patients with OR was 0 (0 – 19) and in patients with NR was 3 (0 – 25). There was a positive correlation between the lack of clinical response and the number of lymph node metastases (*p *= 0.03; Sperman r). The response to neoadjuvant chemotherapy, the type of surgery performed, and the lymph node status in patients with OR and NR are summarized in Table 3.

### Clinical and histopathological characteristics and their relationship with clinical response

We evaluated the association among clinical response, patients' characteristics (patient's age, menopausal status, number of chemotherapy cycles and clinical stage), and histological (histological grade) and immunohistochemical patterns (ER, PR, p53, p21 and HER-2 protein expression). The median age in patients with OR was 51.5 years (range 35 – 67) and in the group of patients NR, the median age was 47.5 years (*p *= 0.3, Student *t *test). In OR group, 24 patients (52.1) and in NR group, 8 patients (57.1%) %) were classified as post menopausal status (*p *= 0.9; Chi-square test). The median number of chemotherapy cycles was three in both groups (*p *= 0.4; Mann-Whitney test) and there was no difference in clinical stage between the OR and NR groups of patients (*p *= 0.78; Chi-square test).

The estrogen receptor was positive in 72.7% of patients with OR and in 85.7% of NR patients (*p *= 0.48; Fisher's exact test) and the progesterone receptor was positive in 44% and 50% of patients with OR and NR, respectively (*p *= 0.76, Fisher's exact test). There were no p53, p21 and HER-2 protein expression differences before chemotherapy between the OR and NR groups (*p *= 0.19, *p *= 1.0 and *p *= 0.43; Fisher exact test, respectively). The histological grade was not able to predict clinical response (p = 0.2; Chi-square test). Table 4 shows the relationship between the clinical response and clinical, pathological and immunohistochemical patterns.

### Neoadjuvant chemotherapy and p53, p21 and HER-2 proteins expression change

The expression of p53, p21 and HER-2 proteins was evaluated in biopsy specimens from 60 breast cancer patients before neoadjuvant chemotherapy. Because no or scarce residual tumor remained after chemotherapy, the evaluation of p53 and p21 protein expression was not possible in 6 patients specimens and, HER-2 protein expression was not carried out in 4 of the patient specimens.

Positive p53 expression was identified in 18 patients (30%) before and in 8 patients (14.5%) after neoadjuvant chemotherapy. There was a significant reduction in p53 protein expression levels after chemotherapy (*p *= 0.039; difference 13.2%; McNemar test). The expression of p21 protein was positive in 13 patients (21.6%) before and in 5 patients (9.2%) after neoadjuvant chemotherapy. Neoadjuvant chemotherapy reduced p21 protein expression levels (*p *= 0.021; difference= 14.81%; McNemar test). Positive HER-2 protein overexpression was observed in 11 patients (18.3%) before chemotherapy. After neoadjuvant treatment, the overexpression of HER-2 protein was observed in seven patients (12.5%). Neoadjuvant chemotherapy was not able to change HER-2 protein expression levels (*p *= 0.12; McNemar test). Table 5 shows immunohistochemical protein expression levels (p53, p21 and HER-2) before and after neoadjuvant chemotherapy.

### P21 protein expression and its relationship with p53 and HER-2 protein expression

We analyzed the relationship between p21 expression and p53 and HER-2 protein expression in pre-chemotherapy biopsy specimens. In forty patients with p53 negative tumors, we found 16 patients with p21 positive protein expression (40%). In patients with positive p53 expression (n = 16), we identified 4 patients with p21 positive tumors (25%). The difference was not significant (Fisher's exact test; p = 0.2).

In eleven patients with HER-2 overexpression, we observed p21 positive protein expression in four patients (36.3%) and, in 49 HER-2 negative patients, the p21 expression was positive in sixteen patients (32.6%). There was no difference in the expression of p21 in such patients (Fisher's exact test; p = 1.0).

### Disease free and overall survival rates

We evaluated the impact of clinical (age, clinical stage, clinical response, type of surgery), pathological (histological grade, axillary lymph node status) and immunohistochemical (ER, PR, p53, p21 and HER-2 protein expression) features on disease free and overall survival. We observed an inverse correlation between clinical response and the risk of death to disease (*p *= 0.02; Spearman r). In a univariate analysis, only Her-2 overexpression and two other factors related to clinical response were related to overall survival: the type of surgery (HR = 0.41, 95% IC = 0.12 to 0.96; *p *= 0.042) and the presence of extensive (more than six positive lymph nodes) residual disease in axillary lymph nodes (HR = 0.35, 95% IC = 0.08 to 0.78; *p *= 0.017).

**Figure 1:**
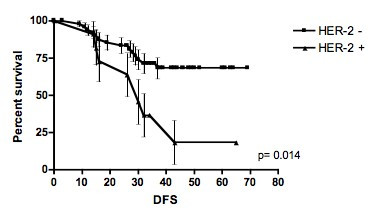
Kaplan-Meyer curve for DFS in sixty patients subjected to Neoadjuvant chemotherapy with docetaxel and epirubicin according to HER-2 protein expression.

**Figure 2:**
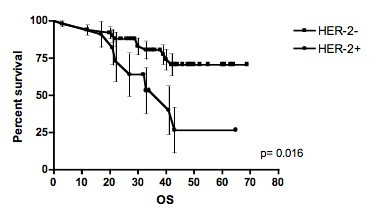
Kaplan-Meyer curve for OS in sixty patients subjected to neoadjuvant chemotherapy with docetaxel and epirubicin according to HER-2 protein expression.

In a multivariate analysis, only HER-2 overexpression was a predictive factor of DFS and OS (*p *= 0.0007 and *p *= 0.003, respectively). The median time to DFS was 31 months (HR = 0.35, 95% IC = 0.08 to 0.75; *p *= 0.014) and the median time to OS was 40 months (HR = 0.33, 95% IC = 0.06 – 0.75; *p *= 0.016). Figures 1 and 2 show the Kaplan-Meyer curve for DFS and OS according to HER-2 protein expression, respectively.

## Discussion

Neoadjuvant or induction chemotherapy is the current standard of care in locally advanced breast cancer [17]. Such treatment can reduce tumor volume, leading to less aggressive surgery and providing favorable local conditions for surgery in inoperable tumors [1,2]. About 80% of patients achieve a local benefit due to adequate tumor downstaging, however, the overall survival, when comparing neoadjuvant and adjuvant chemotherapy, is not influenced by the primary cytotoxic therapy [17].

We evaluated the response to neoadjuvant chemotherapy in 60 locally advanced breast cancer patients. The objective clinical response rate was 76.6%, with a complete pathological response in 5% of patients. Breast conserving therapy was possible in forty patients (66.6%) and in only one patient could the surgery not be performed due to the lack of response to neoadjuvant chemotherapy. Other studies reported a higher complete pathological response rate ranging from 8% to 10.5%, using similar chemotherapy scheme [16,18]. However, the breast conserving surgery rate was higher in our study. They reported a breast conserving surgery rate of 16% and 15%, respectively, in contrast to the study of Ganem *et al *who reported a breast conserving surgery rate of 69% [19]. This discordant result should be attributed to the enrollment of different number of patients with primary inoperable tumors among these studies.

The clinical and pathological response to neoadjuvant therapy in patients with large breast tumors has been described as an independent prognostic factor [3,4]. We observed similar results. In our study, patients who achieved an objective response had a better prognostic than patients with stable or progressive disease after induction chemotherapy. The observation that patients with extensive lymph node metastasis after neoadjuvant therapy are more propitious to develop metastatic disease and die due to disease progression than patients with no extensive axillary metastasis supports the hypothesis that residual loco regional disease after neoadjuvant chemotherapy is a clinical marker of chemotherapy resistance.

The neoadjuvant therapy, considered to be an *in vivo *model to test chemotherapy sensitivity, is helpful to assess biological tumor behavior and to evaluate chemotherapy mechanisms of function and its relationship to drug resistance. In a previous study, we observed that the induction of apoptosis is an important predictor of response to chemotherapy in breast cancer and that such an event is influenced by protein tumor expression patterns [7]. In the present study, we evaluated the correlation among patients' characteristics, immunohistochemical expression of hormonal receptors (ER and PR), p53, p21 and HER-2 protein expression and the clinical and pathological response to a neoadjuvant combination of docetaxel and epirubicin. No parameter studied was able to predict response to neoadjuvant chemotherapy.

Changes in protein expression after neoadjuvant treatment have been reported [20,21]. Such observation suggests that chemotherapy can alter the tumor gene expression and that this event is a important point in drug sensitivity [7]. In the present study, we confirmed the reduction in p53 protein expression, as well in p21 protein expression, after neoadjuvant chemotherapy. However, the taxane and anthracycline combination in a neoadjuvant setting was not able to change HER-2 expression in patients with locally advanced breast carcinoma. The HER-2 stable phenotype during neoadjuvant chemotherapy was reported by others authors [22,23].

Recent studies have been focused on the role of HER-2 as a predictive factor of response to neoadjuvant chemotherapy [24-26]. Zhang F *et al *and Fernández-Sánchez M *et al *could not observe a correlation between HER-2 expression and the clinical or pathological response to neoadjuvant chemotherapy in patients with breast carcinoma treated with 5-fluoruracil, doxorubicin and cyclophosphamide combination (FAC). Otherwise, Learn PA et al demonstrated that the addition of docetaxel to anthracycline-based neoadjuvant chemotherapy improved clinical response rate in HER-2 positive breast cancer patients. In our study, the expression of the HER-2 protein was an independent prognostic factor in multivariate analysis; however, its expression was not able to predict the clinical and pathological primary tumor response.

We observed that the median time of disease free and the overall survival were 30 months and 41 months, respectively, in patients with HER-2 positive tumors. These patients achieved an overall survival rate of 25% after 48 months in contrast with 70% in patients with HER-2 negative expression. According to SEER estimation, patients with clinical stage III and grade 3 breast cancer (T3N1M0), with negative hormonal receptors subjected to no systemic therapy, have a ten year overall survival rate of 33.5% [27]. This observation leads us to offer alternative therapy with target agents to block the HER-2 activity for patients with HER-2 amplification. Recent studies have demonstrated a promising benefit of chemotherapy and immunotherapy (trastuzumab) combination regimens in a neoadjuvant setting in patients with HER-2 amplification breast cancer [28,29].

## Conclusion

We have concluded that the clinical response to neoadjuvant chemotherapy is a relevant prognostic factor in patients with locally advanced breast cancer treated with a neoadjuvant docetaxel and epirubicin combination. The value of biological makers as predictive factor of response to neoadjuvant therapy is still not clear. Advanced breast tumors exhibit a polyclonal population and the high number of genetic dysfunction limits the use of specific markers in clinical practice. Future directions are going to be based on multiple gene expression patterns, called genetic signatures. Recent publications have shown a positive relationship between a gene expression signature and a complete pathological response to primary chemotherapy [30,31].

On the other hand, HER-2 overexpression is the major relevant phenotype in breast cancer progression regardless the clinical response to neoadjuvant chemotherapy. HER-2 overexpression in primary tumor is a stable phenotype during neoadjuvant chemotherapy and is a marker of distant disease progression and death.

## Competing interests

The author(s) declare that they have no competing interests.

## Authors' contributions

DGT participated in the design of the study, recruitment and treatment of patients, draft the manuscript and performed statistical analysis. JMA participated in the design and coordination of the study and helped to draft the manuscript. ARS and MGT performed the immunohistochemical analysis. FEZ and HRCM participated in the treatment of patients and helped with the design of the study. All authors read and approved the final manuscript.

## Pre-publication history

The pre-publication history for this paper can be accessed here:



## Figures and Tables

**Table 1:** Characteristics of sixty breast cancer patients treated with neoadjuvant docetaxel plus epirubicin.

**Characteristcs**	n = 60 (range)	%
**Age**		
Median	50 (29 – 67)	
≤ 45 years	18	30
>45 years	42	70
**Menopausal status**		
Pre	28	46.6
Post	32	53.4
**Chemotherapy cycles (median)**	3 (2 – 5)	
**Clinical Stage**		
IIA	12	20
IIB	19	31.7
IIIA	8	13.3
IIIB	18	30
IIIC	3	5
**Histology**		
Ductal	49	81.7
Lobular	4	6.7
Medular	4	6.7
Outhers	3	5
**Histologic grade**		
I	16	26.7
II	32	53.3
III	11	18.4
**ER**		
Positive	45	75
Negative	15	25
**PR**		
Positive	32	53.3
Negative	28	46.7
**P53 protein**		
Positive	17	28.3
Negative	43	71.7
**P21 protein**		
Positive	20	33.3
Negative	40	66.7
**HER-2**		
Positive	11	18.3
Negative	49	81.7

N = number of patients; ER = estrogen receptor; PR = progesterone receptor.

**Table 2:** Toxic effect incidences in sixty locally advanced breast cancer patients subjected to neoadjuvant docetaxel (75 mg/m^2^) and epirubicin (50 mg/m^2^) combination.

	**Toxicity (%)**
Alopecia	100
Nausea and vomiting	19.2
Neutropenia	11.5
Febrile neutropenia	3.8
Fever	5.7
Diarrhea	5.7
Mucositis	3.8
Allergic reactions	3.8
Asthenia	1.9
Headache	1.9

**Table 3:** Surgery performed in sixty patients with locally advanced breast cancer subjected to neoadjuvant chemotherapy with docetaxel and epirubicin combination.

	**OR N (%)**	**NR N (%)**	***P***	**Test**
**Clinical Response**	46 (76.7)	14 (33.3)	-	-

**Surgery**				
RS	12 (26)	7 (50)		
BCS	34 (74)	6 (42.8)		
NS	0	1 (7.2)	0.03	*x*^2^
**Node**				
Median (range)	0 (0 – 19)	3 (0 – 25)	0.03	Spearman r

OR = objective response; NR = no response; RS = radical surgery; BCS = breast conserving surgery; NS = not submitted to surgery.

**Table 4:** Clinical and histological features of sixty patients subjected to neoadjuvant chemotherapy with docetaxel plus epirubicin. Patients were divided according to clinical response as objective response (OR) and no response (NR) groups.

	**OR (n)**	**NR (n)**	***P***	**Test**
**Age**				
Median (range)	51,5 (35 – 67)	47,5 (29 – 68)	0.3	Student t
≤ 45 anos	12	7		
>45 anos	35	7	0.1	Fisher
**Menopausal Status**				
Post	24	8		
Pre	22	6	0.9	x^2^
**CT cycle (range)**	3 (2 – 5)	3 (2 – 4)	0.4	Mann-Whitney
**CS**				
IIA	8	4		
IIB	15	4		
IIIA	5	2		
IIIB	14	4		
IIIC	3	0	0.78	x^2^
**Grade**				
I	10	6		
II	27	5		
III	8	3	0.2	x^2^
**ER**				
Positive	32	12		
Negative	12	2	0.48	Fisher
**PR**				
Positive	20	7		
Negative	25	7	0.76	Fisher
**ER/PR**				
Positive	16	7		
Negative	22	7	0.75	Fisher
**P53**				
Positive	16	2		
Negative	30	12	0.19	Fisher
**P21**				
Positive	15	5		
Negative	31	9	1	Fisher
**HER-2**				
Positive	10	1		
Negative	36	13	0.43	Fisher

OR = objective response; NR = no response; n = number of patients; CT = chemotherapy; CS = clinical staging; Grade = histological grade (Bloom-Richardson); ER = estrogen receptor; PR = progesterone receptor.

**Table 5:** Immunohistochemical patterns before and after neoadjuvant chemotherapy with docetaxel plus epirubicin in sixty breast cancer patients.

	**Pre-chemotherapy N (%)**	**Post-chemotherapy N (%)**	***p***	**Dif. (%)**
p53 +	18 (30)	8 (14.5)	0.039	13.2
p21 +	13 (21.6)	5 (9.2)	0.021	14.81
HER-2 +	11 (18.3)	7 (12.5)	0.12	NS

N = number of patients; Dif. = difference between before and after chemotherapy – McNemar test; NS = not significant.
